# Cardiogenic Shock Induced by Anterior Pituitary Hypofunction and Thyrotoxicosis

**DOI:** 10.7759/cureus.12347

**Published:** 2020-12-28

**Authors:** Lei Li, Yan Li, Yueqin Gao, Yuyan Hou, Xiaojian Song

**Affiliations:** 1 Department of Endocrinology, Shanxi Cardiovascular Hospital, TaiYuan, CHN

**Keywords:** cardiogenic shock, anterior pituitary hypofunction, thyrotoxicosis

## Abstract

Cardiogenic shock occurs when the heart is unable to pump enough blood for the needs of the body. Hypopituitarism is a condition in which the pituitary gland does not produce enough of one or more hormones, and it rarely occurs with thyrotoxicosis. We report a rare case of cardiogenic shock induced by anterior pituitary hypofunction and thyrotoxicosis. A 47-year-old woman was admitted twice to the hospital due to generalized worsening muscle pain for 13 days, and accompanied by a transient loss of consciousness. Cardiogenic shock developed during hospitalization, which improved with active resuscitative measures. Laboratory tests showed thyrotoxicosis. Pituitary magnetic resonance imaging (MRI) and relevant hormone tests confirmed anterior pituitary hypofunction. The patient was given hormone replacement therapy, which stabilized her condition. We believe cardiogenic shock may be a serious complication of hypopituitarism. We recommend establishing an expert system (ES) to facilitate the early diagnosis and treatment of cardiogenic shock, improve the professional skills of primary care physicians, and optimize treatment plans.

## Introduction

Cardiogenic shock (CS) is an acute physiological condition caused by decreased cardiac output, which can result in inadequate end-organ and tissue perfusion, low blood pressure, and systemic microcirculatory dysfunction. It is a clinical syndrome characterized by ischemia, hypoxia, metabolic disorders, and damage to important organs [1]. Common causes of CS include acute myocardial infarction, severe myocarditis, and massive pulmonary embolism [2]. The occurrence of CS secondary to endocrine disease is rare. Here, we report a rare case of cardiogenic shock induced by anterior pituitary hypofunction and thyrotoxicosis.

## Case presentation

History and physical examination

A 47-year-old female felt generalized muscle pain after getting up on the morning of May 1, 2019. Her body temperature was 37.8°C. On May 7, she began to experience chest pain, pain below the xiphoid process, abdominal distention, nausea, and vomiting. On May 10, she visited a local hospital and was diagnosed with coronary heart disease, for which she was given treatments to inhibit platelet aggregation, stabilize plaques, and dilate coronary arteries. However, the results from the treatments were not satisfactory.

On May 13, the patient suffered a transient loss of consciousness twice, accompanied by urinary incontinence. She regained consciousness after about 3-5 min. She came to our hospital at 6:50 p.m. on the same day. She had a two-year history of hypertension and irregular and light periods for the previous two years. Physical examination at admission showed the following: temperature 36.9°C, pulse 135/min, respiration 30/min, blood pressure 116/88 mmHg, moist and cold skin, and sparse armpit and pubic hair. Moist rales were heard over bilateral lower lungs. The heart border was enlarged on percussion, and her heart rate was 135/min and regular. No obvious pathological murmurs were heard over all auscultatory valve areas. No edema was present in either of her lower legs.

Auxiliary examination

**Figure 1:**
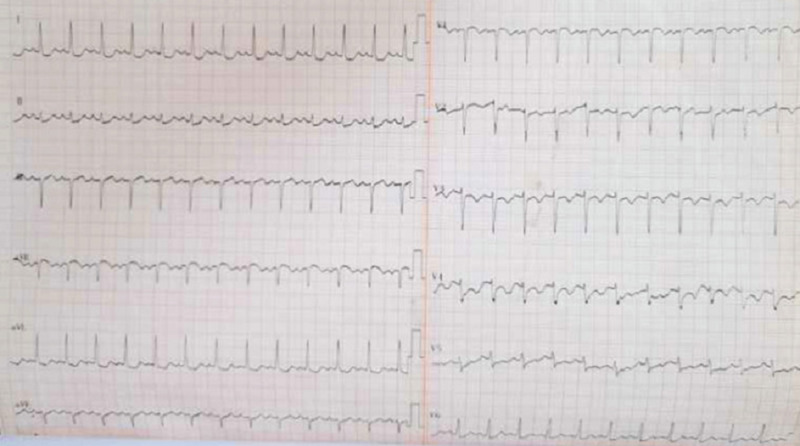
Electrocardiogram (ECG)

The lab tests returned the following results: troponin I 2.87 ng/mL (reference range: 0-0.03); creatine kinase-MB: 16.59 ng/mL (reference range: 0-25); and B-type natriuretic peptide (BNP): 10488.7 ng/L (reference range: 0-125) (Tables 1-2). The electrocardiogram (ECG) showed sinus tachycardia and extensive ST-T changes (Figure 1). Thyroid function tests indicated thyrotoxicosis (Table 3). Color Doppler ultrasound of the heart showed left atrial and ventricular enlargement, left ventricular segmental wall motion abnormalities, decreased wall motion in the middle portion of the interventricular septum and the apex of the left ventricular wall, and decreased left ventricular systolic function (Table 4).

**Table 1:** Changes in blood glucose, blood sodium and myocardial enzymes

Day	Creatine kinase-MB (ng/ml) (reference: 0-25）	Troponin I (ng/ml) (reference: 0-0.03）	Na^+^ (mmol/L) reference: 137-147）	Glucose (mmol/L) (reference: 3.9-6.1）
Day 1	16.59	2.87	141	3.48
Day 3	9	2.50	139	-
Day 5	15	0.53	135	-
Day 14	14	-	138	-
Day 23	16	1.02	140	-

**Table 2:** Changes in BNP levels after admission BNP - B-type natriuretic peptide

Reference range	Day 1	Day 2	Day 4	Day 23	Day 33
0–125 ng/L	10488.7	4662.0	5026.0	2845.0	1802.0

**Table 3:** Changes in the patient’s thyroid hormones and TSH levels after admission TT4 - total thyroxine; TT3 - total triiodothyronine; FT4 - free thyroxine; FT3 - free triiodothyronine; TSH - thyroid-stimulating hormone

Day	TT4 (mg/dL)	TT3 (mg/L)	FT4 (pmol/L)	FT3 (pmol/L)	TSH (mIU/mL)
Day 1	28.54	2.08	72.72	13.98	0.02
Day 7	20.81	1.07	56.70	7.07	0.01
Day 14	11.10	0.56	22.75	4.24	0.04
Day 21	23.97	2.75	54.58	16.54	0.01
Day 32	16.87	1.24	27.45	6.82	0.01
Day 64	8.36	1.47	10.35	5.68	0.02
Day 92	4.99	0.99	6.88	5.58	2.01

**Table 4:** Changes in measured values of heart parameters by color Doppler ultrasound after admission LA - left atrium; LV - left ventricle; RA - right atrium; RV - right ventricle; EF - ejection fraction

Day	LA (mm)	LV (mm)	RA (mm)	RV (mm)	EF (%)
Day 1	37	51	34×42	18	44
Day 3	37	52	28×40	17	43
Day 7	37	50	29×44	19	43
Day 12	33	48	33×41	19	62

Diagnosis and treatment course

Two hours after admission, the patient suddenly showed restlessness and profuse sweating. Her blood pressure dropped sharply to 76/40 mmHg, and cardiogenic shock occurred. An intra-aortic balloon pump was immediately used to provide hemodynamic assistance, and noninvasive ventilation was performed to support breathing. She was also given fluid replacement therapy, after which her symptoms improved, and her blood pressure rose to 114/76 mmHg. Based on the aforementioned test results, she was diagnosed with severe myocarditis, cardiogenic shock, and hyperthyroidism. We formulated the following treatment plan: 1) methylprednisolone 80 mg BID via IV drip; 2) ganciclovir; 3) diuretics; 4) methimazole 10 mg TID. With these treatments, the patient’s symptoms gradually improved, her blood pressure was maintained at 110/70-120/80 mmHg, and her generalized pain also became less intense.

**Figure 2:**
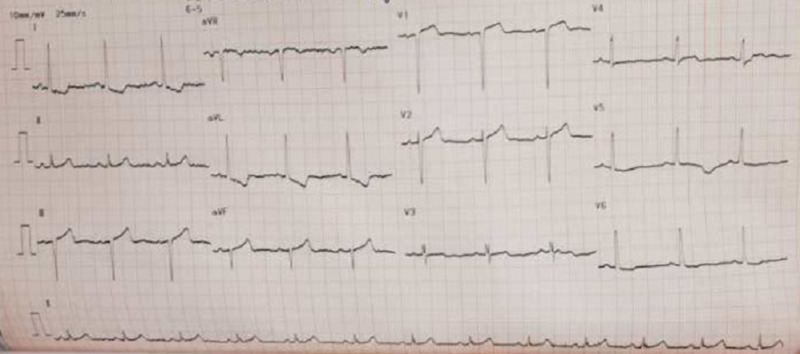
Electrocardiogram (ECG)

**Figure 3:**
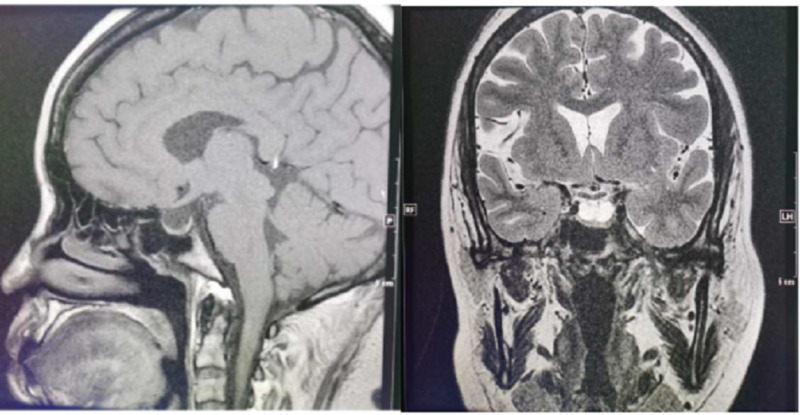
Pituitary magnetic resonance imaging (MRI)

On May 17, the patient became agitated again, with her blood pressure dropping steeply to 42/30 mmHg. The handheld pulse oximeter showed an oxygen saturation of 68%. The intra-aortic balloon pump was again used, which maintained her blood pressure at around 110/80 mmHg and stabilized her condition. On June 3 and 4, the patient suddenly experienced chest pain and decreased blood pressure (92/64 mmHg). ECG showed inverted T-waves returned to the upright position in leads V1-V6 (Figure 2). In order to determine the cause of the disease, we performed a heart MRI, which showed a left atrial diameter of 32 mm, left ventricular diameter of 50 mm, far left ventricular myocardial motion, homogeneous myocardial signals in the black-blood sequences, and fair suppression of blood flow in the heart chambers (see Figure 3).

**Figure 4:**
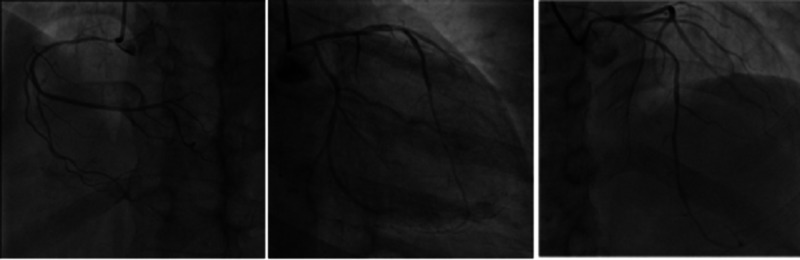
Coronary angiography

We also performed coronary angiography. There was no obvious stenosis of the left main coronary artery, about 75% stenosis in the proximal left anterior descending artery (LAD), about 70% stenosis in the distal LAD, around 90% stenosis in the proximal segment of the first diagonal branch, no obvious stenosis in the circumflex artery, approximately 50%-70% diffuse stenosis between the opening and proximal segment of the right coronary artery, and equal coronary artery dominance (see Figure 4). She was then diagnosed with coronary heart disease (CHD) and was given treatments to inhibit platelet aggregation, stabilize plaques (clopidogrel, 75 mg per day), dilate coronary arteries (atorvastatin calcium tablets, 20 mg per day), and relieve chest pain (nicorandil, 5 mg TID).

Given that the above tests failed to explain the patient’s recurrent hypotension, we assessed her endocrine system. Pituitary MRI showed empty sella turcica. Her hormone test results were as follows: adrenocorticotropic hormone (ACTH) 5.2 ng/L (reference range: 7-64), prolactin 9.86 ng/mL (reference range: 3.34-26.72), follicle-stimulating hormone 45.37 mIU/mL (reference range: 4.54-22.51), luteinizing hormone 24.70 mIU/mL (reference range: 19.18-103.03), progesterone <0.01 ng/mL (reference range: 5.16-18.56), and estradiol 40.00 pg/mL (reference range: 49-291). Based on these test results, she was diagnosed with anterior pituitary hypofunction and was given prednisone tablets 25 mg per day.

Treatment outcome

After prednisone therapy, the patient showed a stable heart rate and blood pressure. She had no chest tightness, chest pain, or fatigue. During the one-week observation period, her condition was stable, and she was discharged from the hospital. She complained of no obvious discomfort at the first, third, and sixth-month follow-ups. The dosage of prednisone was gradually reduced to 5 mg per day.

## Discussion

CS is a physiological state in which inadequate end-organ perfusion results from cardiac dysfunction, and it is caused by decreased cardiac output in the presence of adequate blood volume [3]. The main management strategies for CS include: pump failure in acute myocardial infarction (AMI), severe right ventricular myocardial infarction, end-stage cardiomyopathy, fulminant myocarditis, drug toxicity (negative inotropic drugs, cardiotoxic chemotherapy drugs), severe acid-base imbalance and metabolic disorder, severe infection, and inflammation, congenital heart disease, pulmonary embolism, acute pericardial tamponade, constrictive pericarditis [1, 4]. Despite the progress made in the early diagnosis and treatment of CS, its mortality rate remains very high [5]. The main management strategies for CS are shown in Table 5 [6].

**Table 5:** Recommendations regarding management of patients with cardiogenic shock ICU - intensive care unit; CCU - coronary care unit; ACS - acute coronary syndrome; ECG - electrocardiogram; SBP - systolic blood pressure

Recommendations	Class	level
In all patients with suspected cardiogenic shock, immediate ECG and echocardiography are recommended.	I	C
All patients with cardiogenic shock should be rapidly transferred to a tertiary care center that has a 24/7 service of cardiac catheterization and a dedicated ICU/CCU with availability of short-term mechanical circulatory support.	I	C
In patients with cardiogenic shock complicating ACS, an immediate coronary angiography is recommended (within two hours of hospital admission) with an intent to perform coronary revascularization.	I	C
Continuous ECG and blood pressure monitoring are recommended.	I	C
Invasive monitoring with an arterial line is recommended.	I	C
Fluid challenge (saline or Ringer’s lactate, >200 mL/15–30 min) is recommended as the first-line treatment if there is no sign of fluid overload.	I	C
Intravenous inotropic agents (dobutamine) may be considered to increase cardiac output.	IIb	C
Vasopressors (norepinephrine preferable over dopamine) may be considered if there is a need to maintain SBP in the presence of persistent hypoperfusion.	IIb	B
Short-term mechanical circulatory support may be considered in refractory cardiogenic shock depending on patient age, comorbidities, and neurological function.	IIb	C

It is worth noting that timely and accurate diagnosis of the primary disease is of paramount value to effective treatment. Complex factors led to the condition observed in the present case, which, to some degree, delayed the diagnosis of the primary disease. It reminds us of the importance of monitoring the circadian rhythm of cortisol secretion, measuring adrenocorticotrophic hormone levels, and assessing pituitary function in patients with low blood pressure.

Our patient firstly had viral infection symptoms and then had chest pain and gastrointestinal discomfort one week later. She was misdiagnosed with CHD at the local hospital; CHD is the most common cause of chest pain. Although both CHD and myocarditis can lead to elevated levels of myocardial enzymes, they can be differentiated in the following ways: CHD is often precipitated by physical activity and strong emotions and manifests as sudden precordial pain and localized ST-T changes on ECG. In contrast, myocarditis is often caused by a viral infection, and an ECG would show extensive ST-T changes, with no clear localization of myocardial infarction.

Viral myocarditis is often misdiagnosed as CHD in clinical practice. In 2019, Mosebach et al. reported a case of acute myocarditis presenting as an acute coronary syndrome [7]. In 2020, Tran et al. studied a case of idiopathic eosinophilic myocarditis presenting with features of an acute coronary syndrome [8]. Viral myocarditis is regarded as a precursor of cardiomyopathy, which results from common viral infections and post-viral immune-mediated responses [9]. Recovery of heart function is possible in most patients when the viral infection has cleared. However, chronic remodeling can also happen, leading to permanent cardiomyopathy [9-11]. With antiviral treatment, our patient’s heart function recovered, which is consistent with the outcome of viral myocarditis.

Our patient also had CHD, which can co-exist with viral myocarditis [12]. Such co-existence may result in atherosclerotic plaque destabilization due to the inflammatory processes caused by viruses [13]. The viral infection produces a large number of inflammatory cytokines at the affected site, which enter the blood circulation, causing inflammatory reactions in the blood vessel wall. This explains why patients still have angina when their cardiac function returns to normal. For this patient, we comprehensively evaluated her condition with coronary angiography and initiated aggressive treatment to inhibit platelet aggregation, stabilize plaques, and dilate coronary arteries, which improved her condition.

In addition, our patient had thyrotoxicosis. Studies have revealed the molecular and cellular mechanisms by which thyrotoxicosis alters cardiovascular hemodynamics and results in varying clinical manifestations [14-17]. Thyrotoxicosis can aggravate viral myocarditis. In 2019, Sourial et al. reported a case of thyroid storm-induced severe dilated cardiomyopathy [18]. They confirmed that anti-thyroid drug (ATD) therapy can improve the recovery of the cardiomyopathy by suppressing the thyrotoxic state and correcting the primary hemodynamic disturbances. Therefore, early treatment of thyrotoxicosis made a great difference to our patient’s recovery. The methimazole administered in the early phase of her disease inhibited thyroid hormone synthesis reduced the impact of thyrotoxicosis on the heart muscles, and promoted her recovery from viral myocarditis.

The co-existence of hypopituitarism and thyrotoxicosis is rare. After reviewing her history and physical examination at admission, we found that she had had irregular menstruation in the preceding two years and sparse armpit and pubic hair, both suggestive of abnormal endocrine function. During hospitalization, she showed recurrent hypotension, for which intra-aortic balloon pump support was given twice, and later dopamine was also given to increase blood pressure. This led to our suspicion that she might have had adrenocortical hypofunction. Unfortunately, we did not measure her cortisol and ACTH levels before hormone therapy. In the late phase of her disease, the patient experienced nausea, fatigue, and chest pain again after prednisone was discontinued for two weeks. Her cortisol circadian rhythm was 0.7 mg/dL at 0:00 a.m. (reference range: 6.7-22.6), 6.2 mg/dL at 8:00 a.m. (reference range: 6.7-22.6), and 3.4 mg/dL at 4:00 p.m. (reference range: 0-10). Based on this result and her cranial MRI result, the diagnosis of hypopituitarism was made.

Glucocorticoid deficiency plays an important role in cardiomyopathy. Stress-induced excessive production of catecholamines is toxic to heart muscles, which are left unprotected due to insufficient glucocorticoid, thereby impairing heart function. A deficiency of glucocorticoid also interferes with the transport of membrane calcium pump, affecting myocardial contractility. In 2014, Yoshida et al. reported an unusual case of hypopituitarism and transient thyrotoxicosis following asymptomatic pituitary apoplexy [19]. They believed an immune rebound mechanism due to adrenal insufficiency probably caused the painless thyroiditis. Thyrotoxicosis with adrenal insufficiency poses a high risk for a life-threatening adrenal crisis because thyroid hormones can accelerate the degradation, metabolism, and elimination of cortisol. Therefore, the concurrence of hypopituitarism and thyrotoxicosis would further worsen a patient’s condition.

Through the summary of this case, we hope to have laid the groundwork for establishing an expert system (ES) to facilitate the early diagnosis and treatment of cardiogenic shock [20]. The system can serve as an “extended memory” and “doctor’s assistant,” providing doctors with all kinds of data and common diagnoses and treatment plans to help them solve complex medical problems. It is particularly valuable for helping young and inexperienced doctors improve their diagnostic skills and optimize diagnosis and treatment regimens for their patients.

## Conclusions

Missed and delayed diagnoses of hypopituitarism are common as hypopituitarism often occurs and progresses imperceptibly. Primary disease, such as tumors, trauma, and infection, often makes it more difficult to perceive clinical manifestations of hypopituitarism. Our findings promote the timely diagnosis and early treatment of cardiogenic shock, which we believe may be a severe complication of hypopituitarism. Hypopituitarism should be suspected in patients for whom hyponatremia, hypotension, and hypoglycemia are difficult to correct. In addition, cortisol rhythm, ACTH, and pituitary function tests should be performed in a timely manner. We will continue our work to create an expert database into which we will enter the data of this case so as to provide a reference for the diagnosis and treatment of cardiogenic shock.
